#  Outcomes of a Population-Based Congenital Cytomegalovirus Screening Program

**DOI:** 10.1001/jamapediatrics.2024.5562

**Published:** 2025-01-21

**Authors:** Jessica K. E. Dunn, Pranesh Chakraborty, Emily Reuvers, Lauren Gallagher, Kristin D. Kernohan, Melanie Lacaria, Michelle Barton, Kirk Leifso, Jeffrey M. Pernica, Emeril Santander, Marie Pigeon, Sharon L. Cushing, Johnna MacCormick, Soren Gantt, Stacey Weber, Ari Bitnun, Jason Brophy

**Affiliations:** 1 Newborn Screening Ontario, Children’s Hospital of Eastern Ontario, Ottawa, Canada; 2 Department of Pediatrics, Alberta Children’s Hospital, University of Calgary, Calgary, Alberta, Canada; 3 Department of Pediatrics, Children’s Hospital of Eastern Ontario, University of Ottawa, Ottawa, Ontario, Canada; 4 Children’s Hospital of Eastern Ontario Research Institute, Ottawa, Ontario, Canada; 5 Department Pediatrics, University of Western Ontario, London, Canada; 6 Department of Pediatrics, Queen’s University, Kingston, Ontario, Canada; 7 Department of Pediatrics, McMaster University, Hamilton, Ontario, Canada; 8 Department of Audiology, Children’s Hospital of Eastern Ontario, Ottawa, Canada; 9 Department of Otolaryngology, Head & Neck Surgery, Hospital for Sick Children, University of Toronto, Toronto, Ontario; 10 Department of Pediatric Otolaryngology, Head & Neck Surgery, Children’s Hospital of Eastern Ontario, University of Ottawa, Ottawa, Ontario, Canada; 11 Sainte-Justine University Hospital Research Centre, Montreal, Quebec, Canada; 12 Department of Microbiology, Immunology and Infectious Diseases, University of Montreal, Montreal, Quebec, Canada; 13 Ministry of Children, Community, and Social Services, Toronto, Ontario, Canada; 14 Department of Pediatrics, Hospital for Sick Children, University of Toronto, Toronto, Ontario, Canada

## Abstract

**Importance:**

Detection of congenital cytomegalovirus (cCMV) infection has previously relied on targeted screening programs or clinical recognition; however, these approaches miss most cCMV-infected newborns and fail to identify those infants who are asymptomatic at birth but at risk for late-onset sensorineural hearing loss.

**Objective:**

To determine the feasibility of using routinely collected newborn dried blood spots (DBS) in a population-based cCMV screen to identify infants at risk for hearing loss and describe outcomes of infants screened.

**Design, Setting, and Participants:**

This diagnostic study of a population-based screening program in Ontario, Canada, took place from July 29, 2019, to July 31, 2023. All newborns with a DBS sample collected as part of routine care were screened using polymerase chain reaction (PCR) analysis for cCMV as a risk factor for hearing loss. Infants with positive DBS PCR results for cCMV were referred for confirmation of infection by urine PCR (the gold standard), as well as complete medical and audiologic assessments for sequelae of cCMV infection. Infants with possible or confirmed symptomatic cCMV were referred to pediatric infectious disease specialists for evaluation for potential treatment with valganciclovir.

**Exposure:**

Detection of cCMV by polymerase chain reaction assay on a newborn DBS.

**Main Outcomes and Measures:**

Number of infants with positive screening results successfully retrieved and confirmed to have cCMV and the timeliness of retrieval and symptomatic evaluation.

**Results:**

Of 565 987 infants born in the screening period, 551 034 (97.4%) received cCMV screening on the DBS (45.7% female, 54.3% male). Of these infants, 689 (0.13%) screened positive for cCMV; 601 (87.2%) had cCMV infection confirmed and a complete assessment of sequelae of their congenital infection. Ninety-six infants with completed assessments (16.0%) were deemed to have cCMV symptoms, and 63 of these (65.6%) began valganciclovir treatment. Sensorineural hearing loss was confirmed in 34 of 96 infants (35.4%).

**Conclusions and Relevance:**

This program found acceptable and feasible implementation of a population-based screening program using routinely collected DBS samples, suggesting that it may serve as a template for jurisdictions considering universal cCMV screening. The program had a much lower than expected prevalence of cCMV-positive screens but still identified many children who would otherwise not have been diagnosed and who would benefit from ongoing audiologic surveillance.

## Key Points

**Question:**

Can congenital cytomegalovirus (cCMV) be effectively screened for in a population-based program by using newborn dried blood spot (DBS)–based testing?

**Findings:**

In this diagnostic study of a provincial newborn screening program, 551 034 newborns were screened for cCMV using the DBS collected at birth, and 689 (0.13%) had positive results. Of these, 601 infants were confirmed to have cCMV infection and completed medical and audiologic assessments, with 96 (16%) found to be symptomatic.

**Meaning:**

These results suggest that universal screening for cCMV is acceptable and feasible as a population-based newborn screen on routinely collected DBS to identify infants who would benefit from long-term audiologic and developmental follow-up.

## Teaser

This cohort study presents the experience gained and lessons learned from the first 4 years of a universal screening program for congenital cytomegalovirus infection among newborns in Ontario, Canada.

## Introduction

Congenital cytomegalovirus (cCMV) is the most common congenital infection, with an estimated overall birth prevalence of 0.64%.^1^ cCMV is a major cause of sensorineural hearing loss (SNHL) and neurodevelopmental disability in childhood.^2,3,4,5^ Only 10% to 15% of infants with cCMV are symptomatic at birth, of whom more than 30% have or will develop SNHL.^5,6,7^ Of the 85% to 90% of infants with asymptomatic cCMV, approximately 10% will develop SNHL by 5 years of age.^6,7^ Identification of cCMV cases, symptomatic or asymptomatic, permits close audiologic follow-up, early detection of SNHL, and beneficial interventions during the critical period of speech and language development.^8^ Early identification of and intervention for children with SNHL results in language and cognitive outcomes that approach those of peers with normal hearing.^9,10^ Furthermore, valganciclovir treatment of infants with symptomatic cCMV disease can improve hearing and development.^11^ In addition to essentially all asymptomatic infections, even most symptomatic infections go undiagnosed by clinicians because their presentation is often subtle and nonspecific. Thus, most cases of cCMV are missed in the absence of a screening program.^12,13,14,15^

Universal cCMV screening using newborn dried blood spot (DBS)–based tesing was added to augment the existing Ontario Infant Hearing Program (IHP) in identifying children at risk for hearing loss. The objective of this expanded newborn hearing screening program was to identify infants with risk factors for hearing loss in a timely manner to optimize their speech and language development. Many jurisdictions have implemented “targeted” cCMV screening programs (eg, high-risk populations or newborns with failed results of the hearing screen), but Ontario was the first to screen for cCMV at the population level, allowing prospective identification of those children at risk for hearing loss and implementation of heightened audiologic and developmental surveillance. Herein we present our experience and lessons learned from the first 4 years of our universal cCMV screening program.

## Methods

### Population

Infants born in Ontario between July 29, 2019, and July 31, 2023, who underwent routine DBS-based neonatal screening were eligible for cCMV screening. Ontario’s universal single-sample DBS screening program has been previously described.^16^ Premature infants born at a gestational age of less than 33 weeks or infants who weighed less than 1500 g routinely had repeat DBS sampling at 21 days of age per the Newborn Screening Ontario (NSO) protocol to reassess risk of congenital hypothyroidism, but all conditions (including cCMV) were rescreened.^17^ Prior to March 2020, verbal consent to test the DBS for CMV was obtained while scheduling the IHP-provided neonatal hearing screen. With the interruption of routine hearing screening due to the COVID-19 pandemic, DBS were universally tested for CMV without requiring consent from March 2020 onwards. This pandemic-related change in consent practices provided an opportunity to compare the timeliness of screening for risk factors for hearing loss with distinct consent models. This study followed the Strengthening the Reporting of Observational Studies in Epidemiology (STROBE) reporting guideline for cohort studies.

### DBS Assay

DNA was extracted from two 3.2-mm DBS punches using a standard Tris hydrochloride extraction method. Initial polymerase chain reaction (PCR) analysis was conducted via a custom test developed in our laboratory to detect the CMV genes *UL55* and *UL83*. Samples with any level of CMV detection, or a ribonuclease P (*RNaseP*) cycle threshold at or above 30 (indicating insufficient DNA in the reaction), were automatically retested in a confirmatory assay with probes for *UL55*, *UL83*, and *RNaseP* using DNA derived from 2 new 3.2-mm punches. Samples with clear amplification of both *UL55* and *UL83* were reported as positive; those with weak or discordant amplification were adjudicated and reported as positive or borderline positive (weak repeat amplification), or testing was repeated. Further assay details and primer sequences are detailed in eAppendix 1 in Supplement 1.

### Clinical Management

A provincial cCMV care pathway, including antiviral treatment guidelines, (eAppendix 2 in Supplement 1) was developed based on Canadian and international consensus guidelines.^18,19,20^ Infants who tested positive for cCMV were referred to a community pediatrician for medical assessment and to the IHP for auditory brainstem response testing. The standardized clinical assessment included confirmation of CMV via urine PCR analysis (the gold standard), review of symptoms at birth, assessment for intrauterine growth restriction, physical examination, laboratory investigations (complete blood cell count with differential, measurement of alanine aminotransferase and bilirubin levels), cranial ultrasonography, and ophthalmologic evaluation. To facilitate timely workup completion, confirmatory urine PCR analysis was ordered concurrently with other investigations.

Infants with cCMV confirmed by urine PCR test results and 1 or more abnormalities were referred to a pediatric infectious diseases (ID) specialist to determine whether additional investigations and/or valganciclovir treatment were indicated. Valganciclovir treatment was recommended for infants with central nervous system disease, eye disease, and severe or life-threatening disease. Infants with isolated SNHL or nonsevere symptoms, for which no consensus exists in the literature, were offered treatment on a case-by-case basis at the discretion of the ID physician. All infants with confirmed cCMV were referred for developmental surveillance by a community pediatrician until 5 years of age (eAppendix 2 in Supplement 1). Infants with hearing loss at birth were followed up as per the provincial IHP protocol. Infants with normal hearing at birth were followed up in a heightened IHP audiology surveillance protocol.^21^

### Definitions

Definitive cCMV was defined as a positive DBS PCR result followed by positive urine PCR result (or a positive blood CMV PCR result if urine was not collected or if the urine test result was negative). False-negative DBS was defined as a negative result of DBS CMV screening but with a diagnosis of cCMV established by other testing (amniotic fluid or urine CMV PCR within 3 weeks of birth) subsequently reported to NSO. This included infants who were evaluated because of fetal ultrasonographic findings or postnatal symptoms compatible with cCMV and infants born to mothers who had CMV infection in pregnancy. This was not a formal process and likely represents an underestimate of missed screens. A false-positive DBS was defined as a positive DBS test result but a negative urine CMV PCR result and nonreactive CMV IgG and IgM testing. Indeterminate or inconclusive cases were defined as positive CMV DBS results and reactive CMV IgG testing (or no additional serological testing available) but negative (or unavailable) urine or blood PCR results. This category was created after identifying a small number of false-negative confirmatory urine results. Asymptomatic cCMV was defined as confirmed cCMV but no findings on physical examination compatible with cCMV and normal results of laboratory investigations, neuroimaging, ophthalmologic examination, and hearing at birth. Symptomatic cCMV included all infants with 1 or more abnormalities compatible with cCMV and/or SNHL at birth.

### Statistical Analysis

Eligible births were identified within the Better Outcomes Registry & Network, a provincial birth database.^22^ Screening details, infant demographic characteristics, and diagnostic evaluation findings were recorded and stored within NSO’s laboratory information system. Screening positivity rate throughout the first 4 years of the program, consent rate during the prepandemic period prior to waiving consent, and infant outcomes throughout the screening pathway were reported. Timeliness was summarized graphically with medians and IQRs of age at first medical assessment, categorization of symptoms after completed diagnostic workup, and initiation of treatment. Age distributions were stochastically compared across consent models for each milestone with left-tailed Brunner-Munzel tests.^23^ Study data were analyzed in R, version 4.3.1 (R Project for Statistical Computing).^24^

## Results

### CMV Screen Positives

**Figure 1.**
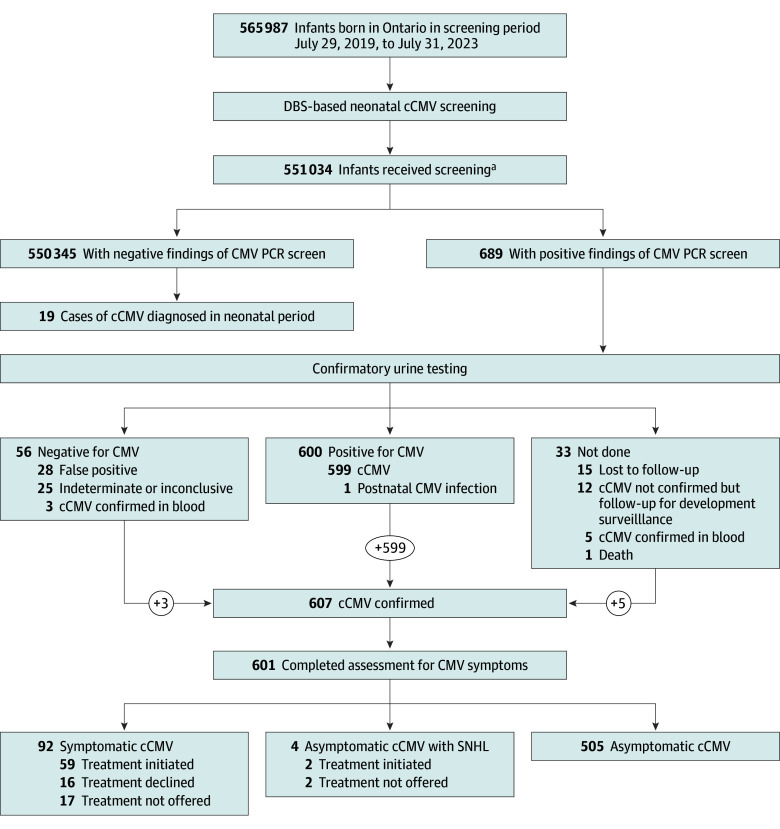
Screening Outcomes cCMV indicates congenital cytomegalovirus; DBS, dried blood spot; PCR, polymerase chain reaction; and SNHL, sensorineural hearing loss. ^a^Reasons for not being screened included being unable to consent for hearing loss risk factor screening (during the prepandemic consented period), inadequate DBS sample, families who declined collection of DBS for all screened diseases, newborn infants who had DBS sampling in another province, those who emigrated prior to DBS collection, palliative care or death, and unknown.

A total 565 987 infants were born in Ontario during the 4-year screening period and 551 034 (97.4%) received cCMV DBS screening (Figure 1). Of these, 689 infants screened positive for cCMV (45.7% female, 54.3% male), resulting in a positivity rate of 0.13%, or 1:800 infants. Stratified annually, this rate was 0.17% in 2019, 0.12% in 2020, 0.10% in 2021, and 0.13% in 2022. Prior to March 26, 2020, 91.1% of infants’ families were approached to consent to screening for routine newborn hearing and risk factors for hearing loss. Of these families, 98.8% agreed to DBS screening for cCMV risk factors. All infants were screened for cCMV after March 26, 2020.

### Medical Assessment of cCMV

Of the 689 infants with positive screen results, all their families were contacted by NSO and 677 (98.3%) were engaged in care, with 578 (83.9%) seen by a community pediatrician or neonatologist for initial assessment. Ninety-nine infants (14.4%) were referred directly to an ID specialist, due to the presence of recognized cCMV symptoms at birth, logistical concerns, or infant hospitalization. Twelve families (1.7%) declined assessment.

Of the 689 infants with positive DBS screen results, 656 (95.2%) had urine collected for confirmation of cCMV infection (Figure 1). Reasons for not having a urine specimen collected among the remaining 33 infants included declined assessment, cCMV confirmation by blood PCR, issues with obtaining or processing the urine sample, referral delayed beyond 6 months, and death (1 infant). Of the 32 living infants without confirmatory urine testing, 15 were lost to follow-up and 17 were followed up for developmental and audiological surveillance (including 5 confirmed to have cCMV by blood PCR results). A total of 601 infants (87.2%) had cCMV infection confirmed and a complete assessment of sequelae of their congenital infection.

Of the 656 infants with urine test results available, 607 DBS-positive infants were confirmed to have cCMV infection (92.5%; 599 by urine PCR and 8 by blood PCR). One premature infant with a negative initial DBS result at day 1 of life but a positive DBS result at 21 days was subsequently deemed to have postnatal CMV infection based on ID consultation. Of the 56 newborns who had negative confirmatory urine PCR results (9.2%), 28 had false-positive DBS results, 25 had results indeterminate or inconclusive for cCMV, and 3 had results confirmed to be cCMV by blood PCR testing.

Of the 601 infants who completed medical and audiologic assessments, 96 (16.0%) were classified as having symptomatic disease; 63 of 96 (65.6%) began valganciclovir treatment. Symptoms occurred either as isolated findings or as multisystem disease. Abnormal physical examination findings (ie, microcephaly, small for gestational age, petechial rash, abnormal neurological examination results, hepatosplenomegaly) were present in 46 of 96 infants (47.9%). The 50 infants (52.1%) with normal physical examination results were categorized as symptomatic based on laboratory, imaging, ophthalmologic, or audiological abnormalities. Laboratory abnormalities were identified in 51 infants (53.1%), and neuroimaging abnormalities were demonstrated in 68 (70.8%) (Table 1). Imaging findings included lenticulostriate vasculopathy (17 [25.0%]), cysts (47 [69.1%]), calcifications (21 [30.9%]), and polymicrogyria (9 [13.2%]), among others. SNHL was confirmed in 34 infants (35.4%), of whom 4 had isolated SNHL. See Table 2 for laterality and degree of SNHL.

**Table 1.** Abnormal Findings in Symptomatic Infants, Occurring as Isolated Findings or as Multisystem Disease

Abnormal finding	Symptomatic infants, No. (%) (n = 96)
Abnormal head ultrasonographic finding	68 (70.8)
Sensorineural hearing loss	34 (35.4)
Intrauterine growth retardation	27 (28.1)
Elevated liver enzyme levels	24 (25.3)
Microcephaly	20 (20.8)
Thrombocytopenia	19 (19.8)
Small for gestational age	9 (9.4)
Conjugated hyperbilirubinemia	8 (8.3)
Abnormal neurologic examination finding	5 (5.2)
Hepatosplenomegaly and/or splenomegaly	4 (4.2)
History of seizures	3 (3.1)
Rash	3 (3.1)
Abnormal ophthalmologic examination findings	2 (2.1)

**Table 2.** Laterality and Degree of SNHL in the Worse Ear

Hearing loss	No. (%) of infants (n = 34)
Ear involvement	
Unilateral	23 (67.6)
Bilateral	11 (32.4)
Degree^a^	
Minimal^b^	5 (14.7)
Mild	4 (11.8)
Moderate	9 (26.5)
Moderate to severe	4 (11.8)
Severe	3 (8.8)
Profound	9 (26.5)

Abbreviation: SNHL, sensorineural hearing loss.

^a^ Degree of SNHL is for the worse ear using the pure tone average from 0.5, 2.0, and 4.0 KHz in decibels of hearing level.

^b^ Minimal loss is used when SNHL is present only at 4.0 KHz in the mild range.

Among the 92 infants with symptomatic cCMV, 77 families were offered treatment with valganciclovir and 61 agreed to antiviral therapy (see eTable in Supplement 1 for treatment indications). Two of the 4 infants with isolated SNHL were offered treatment. The remaining 17 infants were not offered treatment based on mild and/or isolated symptoms.

Twenty-three of the 607 infants with positive screens (3.8%) were identified to have cCMV prior to the DBS result. Of these, 17 infants were classified as symptomatic, with 16 being offered antiviral therapy.

Nineteen infants with negative DBS screens but confirmed cCMV were reported to NSO. These infants were identified based on symptoms of cCMV disease after birth (n = 14), being asymptomatic twins of infants with positive screens (n = 4), or having antenatal findings on fetal imaging but normal assessment at birth (n = 1). Subsequent retesting of these negative DBS screens using the confirmatory assay demonstrated results positive for CMV in 9 infants.

### Timeliness of Screening

**Figure 2.**
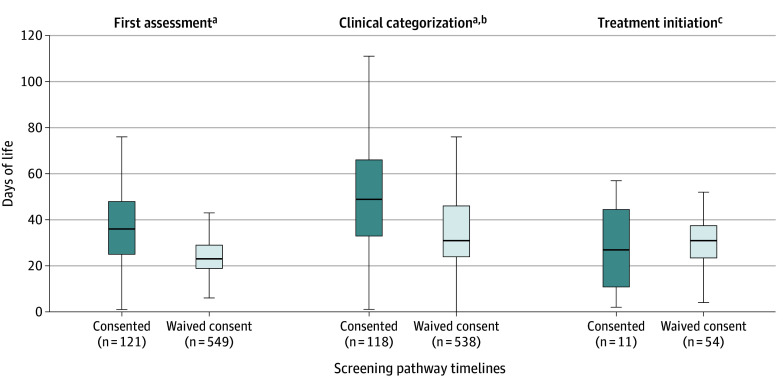
Time to First Clinical Assessment, Completion of Workup and Categorization, and Treatment Initiation Whiskers indicate 150% of the IQR. ^a^*P* < .001, calculated from the Brunner-Munzel test. Significant results support a statistical difference in the frequency of younger ages in the waived consent period for this milestone. ^b^Indicates completion of workup and categorization as symptomatic or asymptomatic. The consented period occurred between July 29, 2019, and March 25, 2020. Waived consent period occurred between March 26, 2020, and July 31, 2023. ^c^*P* = .58, calculated from the Brunner-Munzel test.

During the consented period, 124 infants had positive screens; during the waived consent period, 565 infants had positive screens (Figure 2). Infants screened for cCMV during the waived consent period were assessed and categorized based on symptoms significantly earlier in life than in the consented period. Ages at treatment initiation were not significantly lower in the waived consent period. During the consented period, 1 infant was determined to be eligible for treatment, but it was not offered because the infant was too old (4 months of age) (A. Webster, written communication, February 3, 2020).

## Discussion

From its introduction in July 2019 until July 2023 when these data were compiled, Ontario’s screening program successfully screened 97.4% of infants born in Ontario for cCMV. We found high rates of acceptance during the period of consented testing, with 98.8% consenting to screening for risk factors for hearing loss. This aligns with limited available literature suggesting cCMV screening is positively received, with parents feeling empowered with the knowledge of their child’s health.^25,26,27^ Overall, 677 of 689 infants with positive screens were successfully engaged for initial assessment, further supporting the feasibility of a routine cCMV screening program.

The overall cCMV detection rate of 0.13% in our program (1:800) was lower than the previously reported prevalence rates of 0.2% to 0.5% in high-income countries.^1,28,29^ This may be because cCMV rates in Ontario are lower than those previously reported in other regions, because of low sensitivity of the DBS as a sample type, or both. It is possible that the overall low rate was at least partly related to reduced CMV infection rates due to stringent COVID-19 pandemic restrictions implemented in Ontario, which included prolonged school and daycare closures.^30^ However, this cannot fully explain our results, as the cCMV rate prior to March 2020 (0.17%) was also lower than anticipated. With respect to DBS sensitivity, a systematic review and meta-analysis of prospective studies estimated a pooled DBS sensitivity of 62.3% (95% CI, 54.8%-69.3%).^31^ We decided to use DBS as the screening sample type because of the DBS collection and processing infrastructure already in place. Moving forward, however, the cost and utility of transitioning to saliva or urine screening should be evaluated. Regardless of these limitations, the identification of almost 100 symptomatic and more than 500 asymptomatic infants with cCMV, for whom ongoing hearing and neurodevelopment surveillance has been implemented, is a major accomplishment and a benefit to these children. This is underscored by the fact that only 23 of the 607 infants with cCMV (3.8%) were identified prior to the DBS result. Further, only 17 of the 96 (17.7%) symptomatic infants identified by screening had been identified based on clinical presentation prior to the DBS test results becoming available.

The reporting of 19 cases with cCMV identified on clinical grounds who were not detected by DBS screening was not unexpected, given that the sensitivity of DBS testing is limited when compared with urine or saliva. This highlights the importance of maintaining clinical suspicion for cCMV in newborns with compatible clinical manifestations or antenatal history, regardless of DBS screen results.^12,13^

An unexpected finding was the relatively high rate of negative confirmatory urine testing in those with positive DBS results (9.2%). This is in contrast with the high specificity noted in research settings.^32,33,34^ A single explanation for this finding is unlikely; ad hoc laboratory investigation identified card contamination with CMV DNA on 2 samples, and 2 urine specimens (reported as negative by outside laboratories) had detectable CMV DNA on NSO’s assay that was explained by single nucleotide variants (or polymorphisms) in the primer sequences of the outside assay. However, the explanation for the remainder of these discordant results remains to be determined. A standardized pathway has been implemented for any negative confirmatory urine PCR result that includes referral to an ID physician and reflexive urine CMV culture at a reference laboratory as well as molecular testing of the urine with the NSO assay.

An important clinical management challenge was the numerous children with head ultrasonographic findings of variable severity potentially attributable to cCMV. In particular, the role of magnetic resonance imaging (MRI) and prescribing valganciclovir treatment to infants with isolated neuroimaging findings such as lenticulostriate vasculopathy or isolated cysts was raised by multiple clinicians. Our strategy was to have all such infants reviewed by experienced ID physicians in the context of the infant’s entire workup to assess appropriateness for valganciclovir. Some of these infants underwent MRI at the discretion of the pediatric ID specialist. In some instances, this revealed more severe central nervous system disease, while in other cases, MRI was reassuring. The potential value of MRI, in particular the detection of white matter abnormalities, for predicting disability severity or management of infants with cCMV remains unclear.^35^ Further analysis of our cohort is under way to evaluate the association of nonspecific head ultrasonographic findings with audiologic and developmental outcomes, which may help standardize the role of MRI; in the meantime, we have implemented recommendation of routine head MRI in cases with any head ultrasonographic abnormality. The frequent detection of nonspecific neuroimaging findings in screen positive infants underscores one of our key learnings. Many such infants do not represent the typical symptomatic patient found in traditional cCMV studies and therefore represent a group of infants for whom the potential role of antiviral therapy is uncertain. Most guidelines define symptomatic cCMV based on clinically recognizable findings and therefore do not encompass the more nuanced symptomatology that comes to light in a screening program. As more locales implement universal screening, historic definitions may require revision to account for infants identified via screening who have no clinically evident features but do have abnormalities detected following investigations.

The successful implementation of our cCMV screen can serve as a template for jurisdictions contemplating similar programs. This first program evaluation has demonstrated that it is acceptable to parents, as evident by the high uptake of testing and engagement in care. Our experience reinforces the importance of robust program planning and infrastructure development before rollout. Key highlights include having close programmatic oversight with regular meetings to discuss issues that arise and improve quality; having dedicated staff who contact parents with results and direct their care toward pediatricians or ID physicians as appropriate; engagement of a core group of community pediatricians; and formal educational sessions for all involved in the program. The clinical care pathway that engages community pediatricians for the initial referral establishes a relationship between pediatricians and families and provides continuity with developmental follow-up. This facilitated prompt triaging of symptomatic infants to the ID physicians, keeping referral volume to those clinics manageable. We believe it also decreased the risk of perception of a “medically fragile” child, compared with direct referral to a subspecialty clinic at a pediatric academic center. This program has also raised awareness and better understanding of cCMV infection among pediatric and obstetric care clinicians in the province—a benefit to children, expectant mothers, and their families.

### Limitations

The most important limitation of the program is that, as mentioned, we are unable to quantify how many cases of cCMV were missed using the DBS as the screening modality. To address this, we have initiated a funded study to compare the sensitivity of the DBS with dried saliva samples and to better characterize the prevalence of cCMV in Ontario. Another limitation was incomplete data in a small proportion of cases due to loss to follow-up, lack of engagement by families, or inadequate completion of data report forms by the program clinicians. In addition, as this is a screening program, not a research study, there was some heterogeneity of individual clinician assessments of symptoms and eligibility for antiviral treatment. Likewise, variability in reporting methodology between radiologists with varying degree of pediatric expertise across the province may have impacted interpretation of severity of neuroimaging findings. Last, although available literature supports parental acceptance of cCMV screening,^25,26,27^ there is a knowledge gap on the impact of cCMV screening on families with no sequelae of cCMV infection and those with false-positive results for cCMV, potentially generating undue anxiety for parents.^36^

## Conclusions

This diagnostic study describes the successful implementation of a population-based cCMV screening program over 4 years in Canada’s most populous province. With appropriate planning and engagement of key community and subspecialty pediatric partners, universal newborn screening for cCMV is feasible, acceptable, and beneficial. A substantial number of infants who would not otherwise have been diagnosed with cCMV are now being followed up closely for hearing loss and developmental delay, with the opportunity to intervene early and optimize their outcomes. There remains much to learn about cCMV screening and infants born with cCMV, including the ideal screening modality (eg, DBS vs saliva), the parental experience with screening, outcomes among children with specific symptoms (eg, mild head ultrasonographic abnormalities), as well as the overall long-term audiologic and neurodevelopmental outcomes of infants with cCMV identified by newborn screening.

## Notes

Supplement 1. **eAppendix 1.** CMV DBS Assay Protocol **eAppendix 2.** Newborn Screening Ontario (NSO) Congenital Cytomegalovirus (cCMV) Assessment and Treatment Guidelines **eTable.** Indications for Treatment Among Infants With Symptomatic cCMV Who Received Valganciclovir Therapy

Supplement 2.
Data Sharing Statement
