# Supplementary material for: Outcomes of a Population-Based Congenital Cytomegalovirus Screening Program
Source: JAMA Pediatr. 2025 Jan 21;179(3):332–9. doi: 10.1001/jamapediatrics.2024.5562 (PMC11877178; doi:10.1001/jamapediatrics.2024.5562)
Supplement: Supplement 1. — eAppendix 1. CMV DBS Assay Protocol eAppendix 2. Newborn Screening Ontario (NSO) Congenital Cytomegalovirus (cCMV) Assessment and Treatment Guidelines eTable. Indications for Treatment Among Infants With Symptomatic cCMV Who Received Valganciclovir Therapy [file jamapediatr-e245562-s001.pdf]

Supplementary Online Content

Dunn JKE, Chakraborty P, Reuvers E, et al. Outcomes from a population-based congenital cytomegalovirus screening program. *JAMA Pediatr*. Published online January 21, 2025. doi:10.1001/jamapediatrics.2024.5562

eAppendix 1. CMV DBS Assay Protocol

eAppendix 2. Newborn Screening Ontario (NSO) Congenital Cytomegalovirus (cCMV) Assessment and Treatment Guidelines

eTable. Indications for Treatment Among Infants With Symptomatic cCMV Who Received Valganciclovir Therapy

This supplementary material has been provided by the authors to give readers additional information about their work.

© 2024 Dunn JKE et al. *JAMA Pediatrics*.

eAppendix 1. CMV DBS Assay Protocol

DNA Extraction

A Beckman Coulter SPAN-8 and i5 liquid handler were used to perform automated DNA extraction on all dried-blood spot (DBS) samples. Two 3.2 mm spots of every patient sample were punched into 96-well microtitre plates (Corning 0.360 mL polypropylene round bottom 96 well plate, Fisher). DNA extraction was performed as follows: punches were washed three times with 110 µL of 20 mM Tris-Cl pH 9.0 + 0.5% Triton X-100, followed by one wash with 110 µL of 20 mM Tris-Cl pH 9.0. Plates were incubated at 45 °C and 750 rpm for 10 min between each wash. A total of 50 uL of elution buffer (20 mM Tris-Cl pH 9.0 + 50 ng/µL yeast tRNA) was added to wells. After extraction, the plates were heat sealed (Axygen PlateMax) and incubated at 98°C for 40 minutes and 700 rpm (Vortemp 56 incubator shaker), then centrifuged at 3,000 rpm for 1 min and placed at -20°C until frozen.

Mulitplex qPCR

TRCMV Initial

Multiplex qPCR reactions to detect the presence of CMV and TRECs were performed on ViiA7 qPCR instruments (Life Technologies). The 2X DurAmp Mastermix (Life Technologies) was used with ROX reference dye and four gene specific primers (IDT) and MGB probes (Biosearch) for *UL55* (forward primer 5'-GTTGCCCAACAGGATTTCG-3', reverse primer

5'-CCCGTGGTCATCTTAATTTCG-3' and Cy5-5 probe 5'-CCAGTTGACCGTACTGCACGTACGA-3'), *UL83* (forward primer 5'- GCGCACGAGCTGGTTTG-3', reverse primer 5'- TGGTCACCTATCACCTGCATCT-3' and FAM BHQ Plus probe 5'-CCATGGAGAACACGCGC-3'), TREC (not relevant for this study) and *RNASEP* as a control (forward primer 5'-GCGGAGGGAAGCTCATCAG-3', reverse primer 5'-CCCTAGTCTCAG ACCTTCCCAA-3' and VIC Orange BHQ Plus probe 5'-GCCACGAGCTGAGTGCGT-3'). The Beckman Coulter Biomek NXMC and i7 liquid handlers were used to set up the qPCR reaction. Mastermix was distributed to a 384-well PCR plate (Life Tech Optical Fast) followed by 9.8 µL of extracted DNA for a 20 µL total reaction volume. PCR cycling conditions were as follows: initial denaturation of 95°C x 45 sec, and 45 cycles of 95°C x 45 sec and 60°C x 90 sec.

CMV Confirm

Multiplex qPCR reaction to confirm the presence of CMV were performed as above with the following modifications: TREC primers and probes were removed, 24 µL of extracted DNA was tested in duplicate wells for a 50 µL total reaction volume in 96-well plates (Life Tech Optical Fast).

© 2024 Dunn JKE et al. *JAMA Pediatrics*.

eAppendix 2. Newborn Screening Ontario (NSO) Congenital Cytomegalovirus (cCMV) Assessment and Treatment Guidelines

<u>Congenital CMV Infection – Initial Assessment</u>

Confirmation of diagnosis

- Urine CMV PCR
  - If urine CMV PCR is positive within 3 weeks of birth, a diagnosis of cCMV is confirmed; if for practical reasons the urine PCR is performed later than 3 weeks of age, the diagnosis would still be considered confirmed, but efforts should be made to obtain sample by 3 weeks if possible.
  - If urine CMV PCR is negative, notify NSO and **refer to ID for further testing and interpretation**.

Initial pediatric assessment of symptoms (this assessment should be expedited in order to try to have results before 4 weeks of age):

Physical examination for:

- Anthropometrics (head circumference, weight, length), using WHO growth chart appropriate for sex and gestational age
- Dermatologic: petechial rash, intradermal hematopoiesis (blueberry muffin), jaundice
- Abdominal: hepatomegaly and/or splenomegaly
- Neurological: tone, primitive reflexes

Audiology assessment with auditory brainstem-evoked response (to be arranged by the Infant Hearing Program)

- Referral to otolaryngology and ID if sensorineural hearing loss (SNHL) identified

Imaging:

- Cranial ultrasound for all
- *MRI to be considered in consultation with regional ID Clinic if abnormal findings on ultrasound (if further detail required for diagnosis), if microcephaly is present (to better evaluate for cortex abnormalities) or on case-by-case basis [if MRI is being performed and child has hearing loss, should order both brain and internal auditory canal imaging]. CT is not generally recommended due to radiation exposure.*
- *Abdominal ultrasound to be considered if abnormal findings on physical exam for organomegaly*

Laboratory:

- Complete blood count
- Liver function tests (ALT, direct and total bilirubin)

Ophthalmology:

- As of January 17, 2022, a fundoscopic exam by Ophthalmology is no longer recommended routinely for all CMV screen positive infants. This conclusion was reached after there were no CMV-related eye findings observed in *asymptomatic* infants in the first two years of the program. Only infants with established *symptomatic* cCMV should be referred to ophthalmology.
- Eligibility for ophthalmological assessment to be made by, or in consultation with ID.

Referral to be made to ID Clinic for further assessment and treatment decision-making if any symptoms are present. If there is a questionable finding on initial assessment, please contact ID to determine if a referral is indicated. As part of their assessment, ID will determine whether, and how urgently, an ophthalmological

© 2024 Dunn JKE et al. *JAMA Pediatrics*.

examination is required. ID may arrange ophthalmology on a more urgent basis for cases where the presence of eye findings would impact treatment eligibility.

Treatment

Valganciclovir 16mg/kg/dose PO BID for six months *(treatment of choice)*

- -Recommended to be initiated by 4 weeks of age
- -Decision regarding initiation of antivirals after 4 weeks of age to be made on case-by-case basis in consultation with pediatric ID specialists and following discussion of potential risks and benefits with parents
- -Dose adjustment may be required for select patients, such as infants <32 weeks gestational age, orthose with renal dysfunction (consultation with pharmacist and/or pediatric ID specialist recommended)
- -No strong evidence to treat longer than six month course, however, for severely affected infants, this may be considered on case-by-case basis in consultation with pediatric ID specialist and parental discussion

Ganciclovir IV may be considered in select infants

- -Those with severe disease (hepatitis, critical thrombocytopenia)
- -Those who are not able to tolerate oral medications (eg. premature infants)
- -Those with known poor GI absorption

Indications for Treatment

**TREATMENT RECOMMENDATIONS**	
**Clear evidence for benefit of treatment**	
CNS disease:<br>-Seizures<br>-Microcephaly<br>-Imaging abnormalities associated with CMV CNS disease:calcifications, white matter changes, ventriculomegaly,lenticulostriate vasculopathy, cysts<br><br>*Eye disease*:<br>-Chorioretinitis<br><br>*Severe (life threatening) single or multi-organ non-CNS disease*, e.g. hepatitis, pneumonitis in premature infants	Treat
**Lack of clear evidence for benefit of treatment**	
Isolated Sensorineural Hearing Loss<br>*Isolated or multiple systemic symptoms or signs*, e.g. IUGR with no alternate explanation for growth restriction, thrombocytopenia, hepatitis, jaundice	Some ID experts would recommend treatment. *Treatment to be considered on case-by-case basis with pediatric ID specialist.*
**No evidence for benefit of treatment**	
Infants with cCMV infection but no symptoms (i.e. isolated viremia or positive urine culture)	Do not treat

Follow-up

Symptomatic children on antiviral treatment

-CBC, ALT, Creatinine weekly x 4 weeks

-If normal values, then move to CBC, ALT, Creatinine every 2 weeks x 4 weeks

-If normal values, then move to CBC, ALT, Creatinine monthly for remainder of time on treatment.

-Frequency of above lab monitoring may need adjustment if abnormalities occur.

-Weight check with medication dose adjustment at each monitoring blood work visit, along with general physical and developmental assessment

© 2024 Dunn JKE et al. *JAMA Pediatrics*.

-If severe neutropenia (<0.5), hold valganciclovir for 1 week then reassess with CBC, restart only once neutrophils >0.75-1.0. If significant anemia for age, particularly if rapid drop in hemoglobin is noted, hold valganciclovir and restart only once improved. For severe cases, consider seeking Hematology consultation regarding adjuvant therapies (eg. G-CSF, transfusion).

All cCMV screen positive infants should have the following developmental follow-up by a community pediatrician or pediatric ID specialist:
-Developmental follow up with three assessments in the 1st and 2nd years (including one at 14 and one at 22 months +/- 1 month)
-One assessment in the 3rd, 4th, and 5th years
-Additional assessments as clinically indicated
-Tool: ASQ3 (additional tools (e.g. ASQSE) should be used if felt to be clinically indicated)

Audiology surveillance in accordance with IHP protocol according to symptomatology.
[NOTE: For severe sensorineural hearing loss eligible for cochlear implantation, ID should ensure appropriate vaccination against meningococcus and pneumococcus, including Men-ACYW135 and Men-B series and extra dose of PCV-13 and 2-year PPS.]

DEFINITIONS

Anthropometrics:
• Head circumference: <3rd percentile (adjust for gestational age in premature infants)
• If head circumference centile is significantly lower than height and weight centiles (i.e. relative microcephaly), discuss with pediatric ID specialist

CBC with differential:
• Thrombocytopenia (<150x109/L)

LFTs (ALT, bilirubin):
• ALT: >50 U/L
• Direct bilirubin: 0-14 days >12, over 14 days >5

Head ultrasound findings[1]:
• Calcifications
• White matter changes
• Ventriculomegaly
• Lenticulostriate vasculopathy
• Cysts

[1]Fink et al. Neuroimaging of Pediatric Central Nervous System Cytomegalovirus Infection. RadioGraphics 2010;

© 2024 Dunn JKE et al. *JAMA Pediatrics*.

eTable. Indications for Treatment Among Infants With Symptomatic cCMV Who Received Valganciclovir Therapy

Indications for Treatment with Valganciclovir	Number of Infants Treated (n = 63)
Infants with significant CNS disease only (eg. microcephaly, calcifications, polymicrogyria, white matter hyperintensities, cortical malformations)	10 (16%)
Infants with manifestations of cCMV in two or more systems	49 (78%)
Infants with isolated SNHL	2 (3%)
Infants with mild/non-specific findings	1 (2%)
Infants with non-specific neuroimaging findings	1 (2%)

© 2024 Dunn JKE et al. *JAMA Pediatrics*.
