# Supplementary material for: Outcomes of a Population-Based Congenital Cytomegalovirus Screening Program
Source: JAMA Pediatr. 2025 Jan 21;179(3):332–9. doi: 10.1001/jamapediatrics.2024.5562 (PMC11877178; doi:10.1001/jamapediatrics.2024.5562)
Supplement: Supplement 2. — Data Sharing Statement [file jamapediatr-e245562-s002.pdf]

Data Sharing Statement

Dunn. Outcomes of a Population-Based Congenital Cytomegalovirus Screening Program. *JAMA Pediatr*. Published January 21, 2025. doi:10.1001/jamapediatrics.2024.5562

Data
Data available: No

Additional Information
Explanation for why data not available: Summary data will be readily available, however as the work presented is programmatic data from a screening program and not a consented research study, individual level data will not be shared. Newborn Screening Ontario precludes sharing of individuals' data.
